# *Borrelia burgdorferi* Strain-Specific Differences in Mouse Infectivity and Pathology

**DOI:** 10.3390/pathogens14040352

**Published:** 2025-04-05

**Authors:** Annabelle Pfeifle, Rose Anderson-Duvall, Levi A. Tamming, Wanyue Zhang, Sathya N. Thulasi Raman, Caroline Gravel, Jianguo Wu, Heather Coatsworth, Maarten J. Voordouw, Xu Zhang, Michael J. W. Johnston, Wangxue Chen, Simon Sauve, Lisheng Wang, Xuguang Li

**Affiliations:** 1 Centre for Oncology, Radiopharmaceuticals and Research, Biologic and Radiopharmaceutical Drugs Directorate, Health Products and Food Branch, Health Canada and World Health Organization Collaborating Center for Standardization and Evaluation of Biologicals, Ottawa, ON K1A 0K9, Canada; annabelle.pfeifle@hc-sc.gc.ca (A.P.); rander26@uottawa.ca (R.A.-D.); levi.tamming@hc-sc.gc.ca (L.A.T.); wanyue.zhang@hc-sc.gc.ca (W.Z.); sathya.raman@hc-sc.gc.ca (S.N.T.R.); caroline.gravel@hc-sc.gc.ca (C.G.); jianguo.wu@hc-sc.gc.ca (J.W.); xu.zhang@hc-sc.gc.ca (X.Z.); michael.johnston@hc-sc.gc.ca (M.J.W.J.); simon.sauve@hc-sc.gc.ca (S.S.); 2 Department of Biochemistry, Microbiology and Immunology, Faculty of Medicine, University of Ottawa, Ottawa, ON K1H 8M5, Canada; lisheng.wang@uottawa.ca; 3 National Microbiology Laboratory, Public Health Agency of Canada, Winnipeg, MB R3E 3M4, Canada; heather.coatsworth@phac-aspc.gc.ca; 4 Department of Veterinary Microbiology, Western College of Veterinary Medicine, University of Saskatchewan, Saskatoon, SK S7N 5B4, Canada; 5 School of Pharmaceutical Sciences, Faculty of Medicine, University of Ottawa, Ottawa, ON K1H 8M5, Canada; 6 Department of Chemistry, Carleton University, Ottawa, ON K1S 5B6, Canada; 7 Human Health Therapeutics Research Center, National Research Council of Canada, Ottawa, ON K1N 1J1, Canada; wangxue.chen@nrc-cnrc.gc.ca

**Keywords:** *Borrelia burgdorferi*, Lyme disease, strain-specific differences, infectivity, carditis, lymphadenopathy, tissue abundance, lymph node hyperplasia, antibodies

## Abstract

Lyme disease (LD), caused by infection with the tick-borne bacteria, *Borrelia burgdorferi*, is associated with a wide array of symptoms in human patients. Variations in clinical manifestations are thought to be influenced by genetic differences among *B. burgdorferi* strains. In this study, we evaluated the infectivity, tissue bacterial load, pathology, and immunogenicity of five strains of *B. burgdorferi* sensu stricto (297 Ah130, Bb16-54, B31-A3, Bb16-126, JD1) in female C3H/HeN mice at three infectious doses (10^4^, 10^5^, 10^6^ spirochetes). We found that strains Bb16-126 and JD1 were the most infectious, resulting in 100% infection across all the tested doses. Strain Bb16-126 caused the highest bacterial burden in the heart tissue and significant carditis, whereas JD1 exhibited the lowest spirochete load in the heart and minimal carditis. In comparison, strain B31-A3 demonstrated the highest abundance in the tibiotarsal joint. Infection with all the strains induced severe lymph node hyperplasia, with JD1 producing the greatest increase in cellularity. Using a diagnostic C6 peptide ELISA, all the strains induced significant anti-C6 IgM and IgG antibody titers at 14 days post-infection; however, strain B31-A3 elicited the highest anti-C6 IgM titers. Our findings demonstrate the importance of strain diversity in shaping *B. burgdorferi* pathogenesis in a mouse model and provide insights for developing strain-specific diagnostic, therapeutic, and vaccine strategies.

## 1. Introduction

Lyme disease (LD) is the most common vector-borne disease in the Northern Hemisphere with an annual incidence of nearly 500,000 cases in North America and 650,000 to 850,000 cases in Europe [1,2,3]. LD is caused by spirochete bacteria of the *Borrelia burgdorferi* sensu lato (sl) genospecies complex [4]. In North America, the genospecies that causes the most LD cases is *B. burgdorferi* sensu stricto (Bbss), whereas in Europe, the main genospecies are *Borrelia garinii* in central Europe and *Borrelia afzelii* in North America and Mediterranean Europe [5,6]. These spirochetes are primarily transmitted by ticks of the genus *Ixodes*. The initial infection is localized to the skin, resulting in the characteristic erythema migrans skin rash in 75% of cases [7,8]. If left untreated, the spirochetes can disseminate to colonize the heart, lymph nodes, joints, bladder, and nervous system. Consequently, infection with *B. burgdorferi* can produce a range of severe clinical manifestations in human Lyme disease patients, including arthritis, carditis, and ocular and neurological disorders [9,10,11,12]. Furthermore, *B. burgdorferi* sl genospecies are associated with different disease manifestations: Bbss is associated with arthritis; *B. mayonii* is associated with high spirochaetemia; and *B. bavarensis* and *B. garinii* are more likely to be associated with neuroborreliosis, whereas *B. afzelii* is associated with cutaneous manifestations such as acrodermatitis chronica atrophicans [3,6,13,14,15,16,17,18].

Bacterial pathogens are known to exhibit strain-based differences in infectivity and host responses [19,20,21,22]. Each of the previously mentioned *Borrelia* genospecies can be further subdivided into genetically distinct strains [23,24,25]. In North America, variations in LD symptoms are partly caused by genetic differences among infecting strains of Bbss and from a limited number of *B. Bissettii* and *B. mayonii* infections [26,27,28,29,30]. Experimental infections with different strains of *B. burgdorferi* in rodent models have demonstrated variations in pathology, bacterial burden in host tissues, and immunogenicity [31,32,33,34,35,36,37]. The strain diversity within the Bbss genospecies has been characterized using different strain typing systems including multi-locus sequence typing (MST), the highly polymorphic outer surface protein C gene (ospC), or the ribosomal RNA intergenic spacer type (RST) [38,39,40,41,42,43]. Both *ospC* allele type and RST have been found to influence the infectivity, spirochete dissemination, and resulting arthritis and carditis of *B. burgdorferi* infection [36,44,45]. Furthermore, the *ospC* allele type was shown to influence joint invasion in a mouse model, through the binding of extracellular matrix components [46]. Other studies evaluating strains of Bbss or other genospecies have also demonstrated differences in spirochete loads in the blood, brain, heart, skin, and ankle during infection of both humans and mice [27,35,44,47,48].

Laboratory strains of the house mouse, *Mus musculus*, are the most common animal model for studying Lyme disease [32,36,49,50,51]. The C3H/HeN strain of *M. musculus*, is often used because *B. burgdorferi* infection causes pathology, including arthritis and carditis [52,53,54,55,56]. This mouse model is also used to study host immune responses, antimicrobial testing, preclinical vaccines, and post-treatment Lyme disease syndrome [28,57,58,59,60]. Given the pathogenicity differences between *B. burgdorferi* strains, it is essential to characterize the infectivity and resulting pathology for a variety of strains and at a range of infectious doses in the C3H/HeN mouse model. This remains especially true as new *B. burgdorferi* isolates are collected from the field and novel clones are generated in the lab for use in preclinical research.

In this study, we infected C3H/HeN mice with one of five strains of Bbss at three different doses via needle inoculation. These five diverse strains were isolated from humans and ticks found across the United States in the 1980s or in Canada in the year 2016, and they represent a variety of RST and ospC types (Table 1). First, we determined the infectivity of each strain at each dose. We measured the bacterial burden in the heart and tibiotarsal joint via qPCR and characterized the resulting carditis and lymph node hyperplasia. Finally, we investigated the host antibody response to each strain. Our findings help shed light on strain-specific differences in *B. burgdorferi* infection and pathogenesis.

## 2. Materials and Methods

### 2.1. Bacterial Cell Culture

We used five different strains of Bbss including B31-A3, JD1, 297, Bb16-54, and Bb16-126. Strain B31-A3 was kindly provided by Dr. Patricia Rosa (National Institute of Allergy and Infectious Disease, MD, USA) [63]. Strain B31 was the first isolation strain, filed with the CDC as “ATCC 35210”, where B3 represents the three names of the researchers Barbour, Burgdorfer, and Benach, and the number “1” refers to the first isolation. The suffix “-A3” indicates the clonal derivative of the passage three B31 MI clone. Strain JD1 and strain 297 Ah130 were kindly provided by Dr. George Chaconas (University of Calgary, AB, Canada) [61,62,65,66]. Strain Bb16-54 and Bb16-126 were obtained from the isolate collection of the Public Health Agency of Canada [31,64]. All the *B. burgdorferi* strains were cultured from frozen glycerol stocks at 35 °C and 1.5% CO_2_ in complete Barbour–Stoenner–Kelly (BSK)-H media containing 6% rabbit serum (Millipore Sigma, Burlington, ON, Canada) supplemented with 5 µg/mL of amphotericin B, 100 µg/mL of phosphomycin, and 50 µg/mL of rifampicin. The BSK-H medium is a glutamine-free derivative of CMRL media, which was found to increase spirochete yields of both human and tick isolates [67]. The bacterial cultures were propagated until the mid-log phase, and their density was estimated using a Petroff-Hausser counting chamber under darkfield microscopy. *B. burgdorferi* strains were not sub-cultured to avoid plasmid loss.

### 2.2. Polymerase Chain Reaction (PCR) for Plasmid Characterization

We used the polymerase chain reaction (PCR) to characterize the plasmid profile for each of the five *B. burgdorferi* strains. Cultures for each *B. burgdorferi* strain were grown to mid-log phase and then centrifuged for 15 min at 5000× *g*. DNA was extracted from the pelleted culture using a DNeasy Blood and Tissue Kit (Qiagen, Toronto, ON, Canada) according to the manufacturer’s instructions for Gram-negative bacteria. For each strain, individual PCR reactions were performed for each of 21 plasmids using the primer sets described by Bunikis et al. [68]. Each 50 µL reaction consisted of 10 µL of 5X Phusion HF Buffer (New England Biolabs, Ipswich, MA, USA), 1 µL of 10 mM dNTPs, 2.5 µL each of 10 µM forward and reverse primers, 0.5 µL of Phusion DNA polymerase (New England Biolabs), and 25–50 ng of extracted bacterial DNA. Amplification was performed using an Eppendorf Mastercycler thermal cycler according to the following protocol: initial denaturation for 30 s at 98 °C; 35 cycles of 10 s at 98 °C, 30 s at 60 °C, 45 s at 72 °C; final extension for 10 min at 72 °C. The PCR products were separated by electrophoresis at 200 V for 90 min in a 3% (*w*/*v*) agarose gel in 1X SYBR buffer alongside a 100 bp TrackIT™ DNA ladder (ThermoFisher Scientific, Waltham, MA, USA).

### 2.3. Animal Care and Infectious Challenge

Mice were purchased from Charles River, Senneville, Quebec, Canada. All the animal procedures were approved and performed in accordance with institutional guidelines by the Animal Care Committee at Health Canada (Study #2023-003). Groups of 14-week-old female mice were injected subcutaneously between the shoulder blades with 100 µL of one *B. burgdorferi* strain at one of three infection doses, 10^4^, 10^5^, or 10^6^ spirochetes (*n* = 5 mice per dose per strain). Additionally, five mice were injected with PBS instead of bacterial culture as a negative control (*n* = 5 mice × 3 doses × 5 strains + 5 PBS = 80 mice total). Prior to injection, the bacterial cultures were grown to the mid-log phase, centrifuged at 5000× *g* for 15 min, and diluted in incomplete BSK-H media without rabbit serum. Fourteen days after spirochete injection, the mice were euthanized for the collection of blood and tissues.

### 2.4. Tissue Culture for Confirmation of Infection

Cultures of live *B. burgdorferi* from the host tissues confirmed that the spirochetes were viable. The right ear was collected at euthanasia and cultured at 35 °C and 1.5% CO_2_ in complete BSK-H media containing 6% rabbit serum and supplemented with 5 µg/mL of amphotericin B, 100 µg/mL of phosphomycin, and 50 µg/mL of rifampicin. After 28 days, the samples were examined by darkfield microscopy. Cultures were considered positive for infection if at least one motile spirochete was identified across five fields of view.

### 2.5. Quantitative PCR (qPCR) for Sprirochete Burden

We used quantitative PCR (qPCR) to determine the spirochete burden in the mouse tissues infected with each of the three doses of the five *B. burgdorferi* strains. The left tibiotarsal joint and inferior half of the heart were collected at euthanasia and frozen in liquid nitrogen. DNA extraction and qPCR analysis was performed as previously described [59].

### 2.6. Determination of Lymphadenopathy

Infection with *B. burgdorferi* in mice results in lymph node hyperplasia, which is the proliferation of lymphocytic cells in the lymph nodes. The left axillary lymph node was collected at euthanasia and placed in cold RPMI media supplemented with 0.15% (*w*/*v*) sodium bicarbonate, 1 mM sodium pyruvate, 5 mM HEPES, 20 U/mL of penicillin, 0.02 mg/mL of streptomycin, 0.1% (*v*/*v*) 2-mercaptoethanol, and 10% (*v*/*v*) heat-inactivated fetal bovine serum (FBS). Single-cell suspensions and white blood cell counts were generated as previously described [59,60].

### 2.7. Histopathology

At euthanasia, the heart and right axillary lymph node of each mouse were collected in formalin for histopathology analysis. The tissue processing, hematoxylin and eosin staining, and scoring were performed as previously described [59,60]. In brief, each heart was assigned a numeric grade (0–5), and each lymph node was assigned three numeric grades (0–5) each indicating the severity of follicular hyperplasia, paracortical hyperplasia, or medullary cord plasma cell hyperplasia, respectively. The numeric grade was based on the severity of inflammation where 0 was normal; 1 was minimal; 2 was mild; 3 was moderate; 4 was marked; and 5 was severe.

### 2.8. Enzyme-Linked Immunosorbent Assay (ELISA)

We used ELISA to determine the strength of the mouse IgM and IgG antibody response to the variable major protein (VMP)-like sequence E (VlsE) C6 peptide, which is a commonly used *B. burgdorferi* antigen [69,70]. Blood was collected 14 days after spirochete infection by cardiac puncture exsanguination and centrifuged for 2 min at 10,000× *g* to collect the serum. The wells of 96-well Nunc Maxisorp™ flat bottom plates (ThermoFisher) were coated with 0.1 µg/mL of VlsE C6 peptide (Alpha Diagnostics International, San Antonio, TX, USA) in carbonate buffer pH 9.6 overnight at 4 °C. The plates were washed three times with phosphate-buffered saline (PBS) containing 0.05% Tween-20 (PBS-T). For the IgG ELISA, the plates were blocked for 1 h with 1% (*w*/*v*) bovine serum albumin (IgG-Free, Protease-Free, Jackson Immuno Research, West Grove, PA, USA) in PBS-T. For the IgM ELISA, the plates were blocked for 1 h with 5% (*w*/*v*) milk powder (Rockland Immunochemicals, Limerick, PA, USA) in PBS-T. Mouse serum obtained 14 days after bacterial challenge was two-fold serial diluted in blocking buffer from 1:10 to 1:20,480 and added to the plate. The plates were washed six times with PBS-T and then incubated with HRP-conjugated goat anti-mouse IgG (Cytiva, Marlborough, MA, USA) diluted 1:5000 or HRP-conjugated goat anti-mouse IgM (ThermoFisher) diluted 1:3000 in blocking buffer for 1 h at 37 °C. The plates were washed six more times in PBS-T, incubated for 5 min with 100 µL tetramethylbenzidine (TMB) substrate (Cell Signaling Technology, Danvers, MA, USA), and the reaction was stopped with 0.16 M sulfuric acid. Absorbance was read at 450 nm using a spectrophotometer. Endpoint titers were calculated as the reciprocal of the highest dilution that resulted in an optical density (OD) greater than the average OD of all wells containing serum from control mice injected with PBS plus three times the standard deviation.

### 2.9. Multiplex Cytokine ELISA

Blood was collected 14 days after spirochete infection by cardiac puncture exsanguination and centrifuged for 2 min at 10,000× *g* to collect the serum. Cytokine concentrations in the serum were determined using a ProcartaPlex™ Mouse Th1/Th2 11-plex Cytokine Panel (EPX110-20820-901, ThermoFisher) according to the manufacturer’s instructions. A Luminex 200 system (Millipore-Sigma) was used to read the plate, and data analysis was performed using MILLIPLEX Analyst software (v5.1) (Merck Millipore, Burlington, MA, USA).

### 2.10. Statistical Analysis

Statistical analysis was only performed for the groups injected with 10^6^ spirochetes in which all the strains were found to be 100% infectious. Statistical significance was calculated using an ordinary one-way ANOVA with Tukey’s multiple comparison test. For ELISA data, values were log10-transformed prior to statistical analysis. All the statistical analyses were performed using GraphPad Prism 9 (Dotmatics Inc, Boston, MA, USA) at α = 0.05; * *p*-value  <  0.05, ** *p*-value  <  0.01, *** *p*-value  <  0.001, **** *p*-value  <  0.0001.

## 3. Results

### 3.1. Strains Bb16-126 and JD1 Are Highly Infectious in C3H/HeN Mice

**Figure 1:**
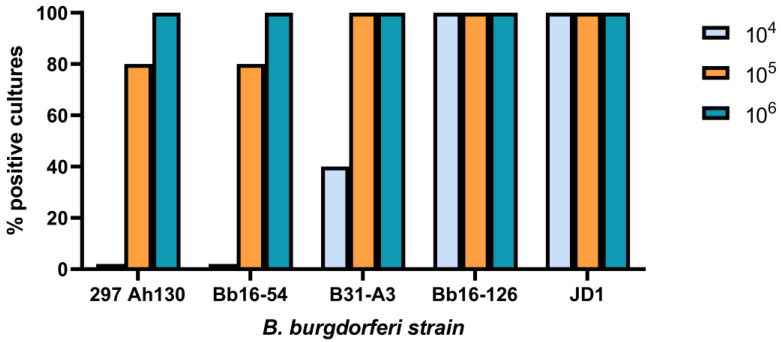
The five *B. burgdorferi* strains differed in their infectiousness in female C3H/HeN mice at 14 days post-challenge. Mice were infected via subcutaneous needle injection with an infectious dose of either 10^4^, 10^5^, or 10^6^ spirochetes. Strains Bb16-126 and JD1 were the most infectious. Mouse ears were collected 14 days after the infectious challenge and incubated for four weeks in BSK-H growth media. Cultures were considered positive if at least one spirochete was identified by darkfield microscopy across five fields of view. Five mice were tested for each of the 15 combinations of strain and dose.

To evaluate the infectivity of the *B. burgdorferi* strains, mice were injected subcutaneously with 10^4^, 10^5^, or 10^6^ spirochetes of one of five strains: 297 Ah130, Bb16-54, B31-A3, Bb16-126, and JD1. Prior to injection, the plasmid content of each strain was assessed using PCR (Table 2, Figures S1–S5). Two weeks after injection, the mice were euthanized, and a range of tissues were collected to evaluate the presence and burden of *B. burgdorferi* infection. We have previously shown that cultivating spirochetes from the ear is a reliable measure of infection at this time point [59,60]. Cultures of live spirochetes from the right ear indicated that Bb16-126 and JD1 were the most infectious strains, both causing 100% infection at all the tested doses (Figure 1). The B31-A3 strain was only 40% infectious at the 10^4^ dose; however, both the 10^5^ and 10^6^ doses were 100% infectious. Finally, the Bb16-54 and 297 Ah130 strains were the least infectious with no infection at the 10^4^ dose, 80% infection at the 10^5^ dose, and 100% infection at the 10^6^ dose. No spirochetes were observed in the ear biopsies from the control mice inoculated with PBS.

### 3.2. B. burgdorferi Strains Differ in Spirochete Load in Mouse Tissues

**Figure 2:**
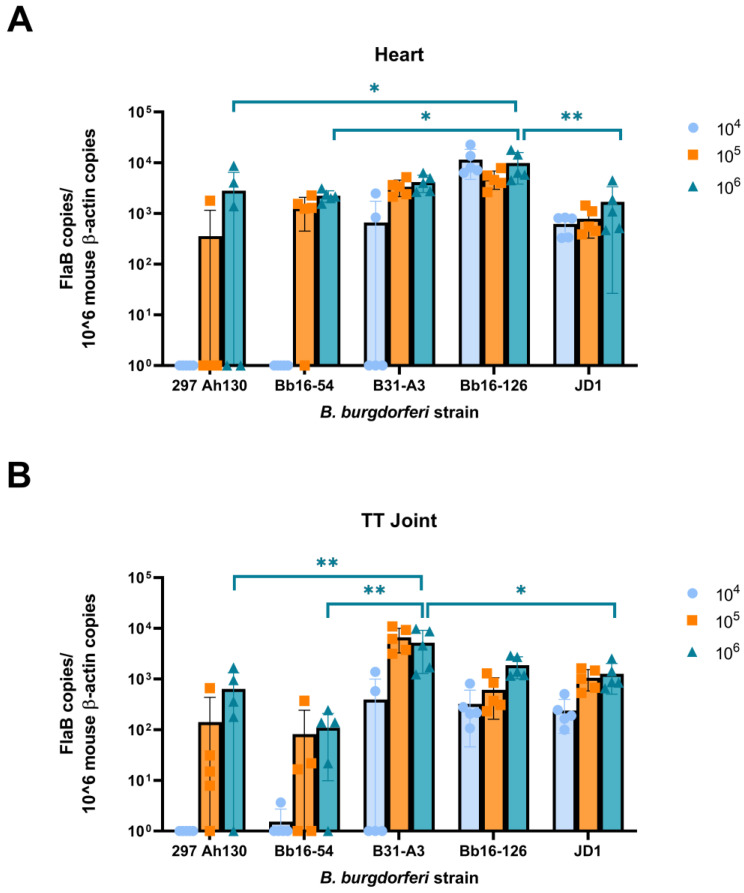
The five *B. burgdorferi* strains differed in their tissue spirochete loads in the (**A**) heart and (**B**) tibiotarsal joint at 14 days post-challenge. Mice were infected via subcutaneous needle injection with an infectious dose of either 10^4^, 10^5^, or 10^6^ spirochetes. Spirochete load was determined by qPCR and has units of the number of copies of the *B. burgdorferi* FlaB gene per million mouse β-actin gene copies. Samples below the threshold of amplification were assigned a value of one. TT joint, tibiotarsal joint. Error bars represent the standard deviation; * *p*-value < 0.05, ** *p*-value < 0.01.

Next, we quantified the bacterial burden in the heart and tibiotarsal joints of the mice using qPCR. The spirochete loads were calculated as the number of *Borrelia FlaB* gene copies per million mouse β-actin gene copies. At the 10^6^ dose, at which all the strains were 100% infectious, all the groups had significant spirochete burdens in both the heart and joint (Figure 2). One of the highly infectious strains, Bb16-126, had the highest mean spirochete load in the heart tissue (Figure 2A). In comparison, the other highly infectious strain, JD1, had the lowest mean spirochete load in the heart tissue. When evaluating the tibiotarsal joint, the highest spirochete loads were observed in mice infected with the B31-A3 strain, while the Bb16-54 strain had the lowest spirochete loads (Figure 2B). None of the control mice inoculated with PBS tested positive for *B. burgdorferi*. Taken together, these data support previous reports that *B. burgdorferi* strains differ in their abundance in host tissues [31,35,37].

### 3.3. Strain JD1 Causes Minimal Carditis in C3H/HeN Mice

**Figure 3:**
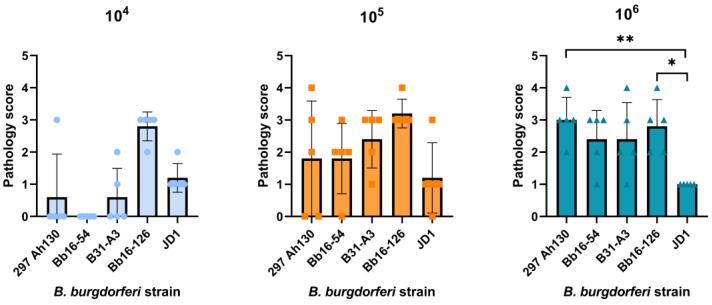
The *B. burgdorferi* strain and infectious dose influenced carditis in the mouse heart. Strain JD1 caused the lowest levels of carditis of all tested strains at the highest infectious doses (10^5^ and 10^6^ spirochetes). Summaries of the carditis pathology scores from heart tissue collected at 14 days post-challenge are shown. Scoring criteria are described in the Section 2 of this article. Error bars represent the standard deviation; * *p*-value < 0.05, ** *p*-value < 0.01.

**Figure 4:**
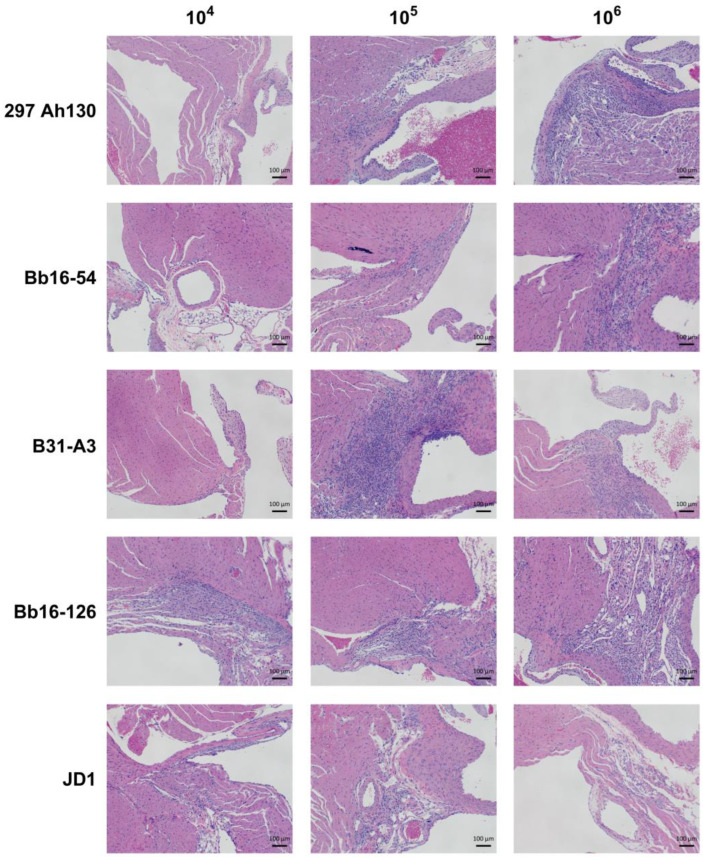
Infection with *B. burgdorferi* induced carditis in the mouse heart. Representative images of H&E-stained heart tissue from mice injected with each of the five *B. burgdorferi* strains at infectious doses of 10^4^, 10^5^, or 10^6^ spirochetes. Scale bar represents 100 µm.

Based on the differences among strains in *B. burgdorferi* burdens in the heart tissue, we evaluated the carditis resulting from infection with each strain. Consistent with the spirochete load in the heart tissue, the Bb16-126 strain caused moderate carditis at all three infectious doses, while the JD1 strain yielded minimal carditis at all doses (Figure 3 and Figure 4). At the 10^6^ dose, at which all the strains were 100% infectious, the 297 Ah130, Bb16-54, and B31-A3 strains all induced moderate carditis. None of the control mice inoculated with PBS had significant levels of carditis. These results suggest that although carditis is a consistent manifestation of *B. burgdorferi* infection, the severity of the inflammation may be strain dependent.

### 3.4. All B. burgdorferi Strains Cause Severe Lymph Node Hyperplasia

**Figure 5:**
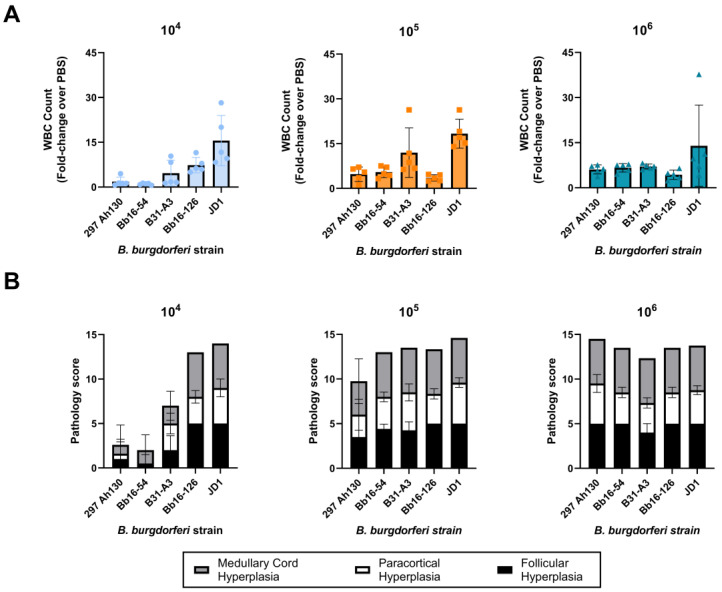
All five *B. burgdorferi* strains induced severe lymph node hyperplasia at 14 days post-challenge. (**A**) White blood cell (WBC) counts from left axillary lymph nodes shown as fold change compared with uninfected control mice inoculated with PBS. (**B**) Summaries of lymph node hyperplasia pathology scores are shown. Scoring criteria are described in the Section 2 of this article. Error bars represent standard deviation.

**Figure 6:**
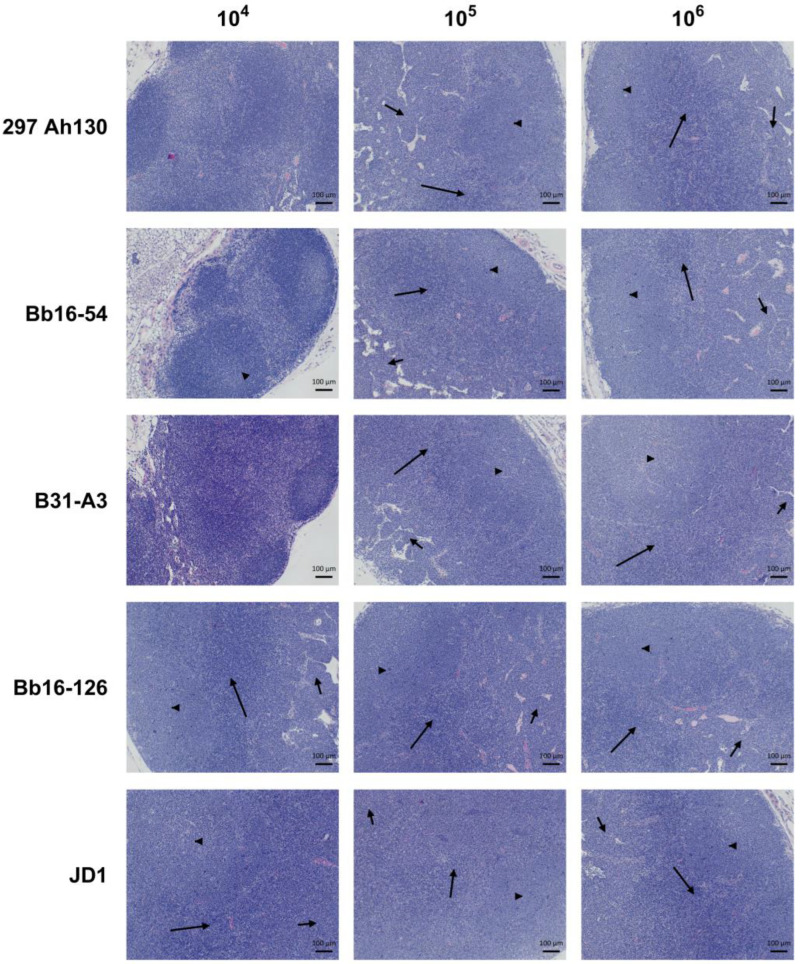
Representative images of H&E-stained axillary lymph nodes from mice inoculated with each of the five *B. burgdorferi* strains at infectious doses of 10^4^, 10^5^, or 10^6^ spirochetes. Arrowheads indicate follicular hyperplasia; short arrows indicate medullary cord hyperplasia; and long arrows indicate paracortical hyperplasia. Scale bar represents 100 µm.

Infection with *B. burgdorferi* is known to cause an accumulation of B cells in the lymph nodes, especially those proximal to the site of infection [71,72,73]. Therefore, we performed white blood cell counts of the axillary lymph nodes at 14 days post-infection for each strain. All the strains induced severe lymph node swelling at the 10^6^ dose, with greater than 4-fold increases in WBC counts compared with mice injected with PBS (Figure 5A). Strain JD1 induced the most severe lymph node hyperplasia, with an average fold change of 14 compared with control mice injected with PBS. Histopathology was also performed on the axillary lymph nodes to further characterize the follicular, paracortical, and medullary cord hyperplasia. At the 10^6^ dose, all the strains induced severe medullary cord hyperplasia marked by dense populations of plasma cells. All the strains also induced severe follicular hyperplasia and moderate-to-severe paracortical hyperplasia marked by dense populations of lymphoid cells in these compartments (Figure 5B and Figure 6). These results suggest that lymphadenopathy is a consistent feature of Bbss infection.

### 3.5. All B. burgdorferi Strains Induce Significant Host Immune Responses

To characterize the host immune response to infection with each strain, we measured the concentration of 11 different cytokines in the mouse serum at 14 days post-infection. High-dose infection with all the strains elicited significant increases in GM-CSF, IL-1β, IL-2, IL-4, IL-5, IL-6, and IL-12p70 when compared with the PBS control (Figure S6). All strains, except Bb16-126, induced IL-18, while none of the strains induced significant IL-13 or TNF-α levels. One of the least infectious strains, 297 Ah130, was the only strain that induced significant increases in serum IFN-γ. It also induced the highest levels of GM-CSF, IFN-γ, IL-6, and IL-18, when compared with the other tested strains. This finding may suggest that certain less infectious strains better stimulate the early host immune response.

**Figure 7:**
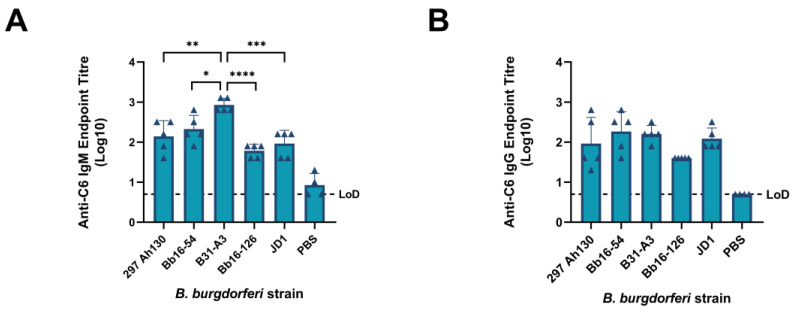
Serum antibody responses to high-dose *B. burgdorferi* infection by strain. ELISA determination of C6-specific (**A**) IgM or (**B**) IgG antibody titers in serum of mice at 14 days post-challenge with a dose of 10^6^ spirochetes per strain. Dashed line indicates the limit of detection (LoD). Samples with data points on the dashed line have antibody titers below the limit of detection. All infected groups had antibody titers that were significantly higher than those in the PBS control group (all *p*-values < 0.01); * *p*-value < 0.05, ** *p*-value < 0.01, *** *p*-value < 0.001, **** *p*-value < 0.0001.

Finally, diagnosis of *B. burgdorferi* infection in humans and canines can be performed by measuring the combined IgG and IgM antibody response to the C6 peptide of the variable major protein (VMP)-like sequence E (VlsE) lipoprotein of *B. burgdorferi*. Convalescent human and canine serum samples can be collected for testing as early as two weeks after the onset of symptoms [74]. Therefore, we measured the anti-C6 IgM and IgG serum antibody titers 14 days after the high-dose infection with each strain. Infection with all the strains induced C6-specific IgM and IgG serum antibody titers that were significantly higher than those in the PBS control group (all *p*-values < 0.01) (Figure 7). This finding suggests that infection would be identifiable using the C6-diagnostic assay as early as 14 days after high-dose infection with all the tested strains. In addition, mice infected with the B31-A3 strain had significantly higher C6-specific IgM titers than those with the other four strains, whereas no differences between strains were observed for anti-C6 IgG titers at 14 days post-infection. Therefore, C6-based assays may more easily detect early infection with specific strains, such as B31-A3.

## 4. Discussion

In this study, we compared five *B. burgdorferi* strains based on the following phenotypes: (1) infectivity, (2) spirochete load in the tibiotarsal joint and heart, (3) carditis, (4) lymph node hyperplasia, and (5) host antibody response. We evaluated Bbss from two Canadian tick isolates (Bb16-54 and Bb16-126) and three derivatives of common laboratory strains (297 Ah130, B31-A3, JD1 Clone SK143) that represent a variety of RST and *ospC* types. Furthermore, the infectivity and tropisms of many of these strains have not been comprehensively characterized in the literature to date.

Previous studies examining the pathogenicity of the JD1 and Bb16-126 strains are limited. JD1 was found to be 100% infectious in rhesus macaques, causing erythema migrans, conjunctivitis, and inducing IgM and IgG antibodies [75]. Bb16-126 was determined to be 100% infective in mice at a dose of 10^4^ or 10^5^ spirochetes and resulted in similar tissue loads to other isolates from the same collection [31]. Here, we found that JD1 and Bb16-126 were the most infectious of the five strains we tested, causing 100% infection in female C3H/HeN mice at the lowest tested dose (Figure 1). Infection with Bb16-126 also resulted in the highest spirochete burden in the heart (Figure 2A). Clinically, two different strains of Bbss have been isolated in culture from a patient with myocarditis: Myo I and Myo II [76]. All the strains in our study were found to cause mild-to-moderate carditis at the highest dose, except JD1, which consistently produced the lowest carditis scores (Figure 3 and Figure 4). These results are in line with JD1 infection generating the lowest spirochete load in the heart tissue. In contrast, JD1 was found to induce the highest-fold increase in lymph node cellularity. However, all the strains consistently induced severe lymphadenopathy (compared with the uninfected control mice) characterized by hyperplasia of the lymph node paracortex, medulla, and follicles (Figure 5 and Figure 6).

Strains B31-A3 and Bb16-54 had the highest and lowest spirochete load in the tibiotarsal joint, respectively (Figure 2B). The preferential joint tropism of strain B31-A3 is consistent with the findings of Sertour and colleagues, which demonstrated that B31 was among the most prevalent strains in the ankle joint and skin at 38 days post-infection via needle inoculation when compared with other *B. burgdorferi* strains [35]. Despite the high abundance of spirochetal DNA in the tibiotarsal joint, we did not detect any significant arthritis at 14 days post-infection for any of the five strains by both measurement of the tibiotarsal joint diameter and histology analysis. Numerous studies have shown that infection with *B. burgdorferi* induces ankle swelling in mice, but peak ankle swelling generally occurs between weeks four and six post-infection [36,51,54,55,56,77,78,79]. As we euthanized the mice in this study at two weeks post-infection, it may have been too early to observe the development of arthritis.

Finally, we found that infection with all the five strains of Bbss induced significant host IgM and IgG antibody responses to the conserved C6 peptide at 14 days post-infection (Figure 7). The C6 immunoassay is frequently used for the diagnosis of LD in canines and previously comprised the first tier of the two-tiered LD diagnostic test for humans [80,81,82]. We found that strain B31-A3 elicited a significantly higher anti-C6 IgM response than all the other tested strains, suggesting it may be more easily detected during early stages of infection using the C6 diagnostic method than the other strains.

The observed strain-specific differences in infectivity, spirochete load, pathology, and immunogenicity are likely due to genetic differences between strains. The genetic makeup of *B. burgdorferi* is highly complex, consisting of an approximately 950 kb linear chromosome and a variable set of circular and linear plasmids (cps and lps) [40]. Of the 21 plasmids that we evaluated, 10 were present in all five tested strains (cp26, cp32-1, cp32-7, lp17, lp21, lp28-1, lp28-2, lp28-3, lp28-4, lp54) and one was absent in all the strains (lp5). As expected, the conserved plasmids included lp54, cp26, and lp28-1, which encode the virulence factors OspA, OspC, and VlsE, respectively [83,84,85]. Three other “core” plasmids, lp28-4, lp28-3, and lp17, were also present in all the strains [86]. Although lp28-4 and lp28-3 are not required for infection, loss of either plasmid is associated with reduced infectivity [87,88,89]. Furthermore, lp17 was recently shown to be required for disseminated infection, evasion of the host adaptive immune system, and the induction of arthritis and carditis [90]. Despite previously being shown to not be required for mouse infection, we also detected lp21 and lp28-2 in all five strains [87]. Lp28-2 is thought to encode a putative replicative DNA helicase [90,91]. The function of lp21 is unknown; however, it has been reported to encode the largest known prokaryotic repeat tract [92]. Two additional circular plasmids, cp32-1 and cp32-7, were also detected in all the strains and have previously been shown to be well-retained across isolates [86,87,93]. Only the smallest plasmid, lp5, was absent in all the strains. This plasmid is known to be lost by *B. burgdorferi* at a high frequency [93].

Four linear plasmids and six circular plasmids were variably present in the five evaluated strains. One of the least-infectious strains, 297 Ah130, possessed the fewest plasmids, with only seven linear plasmids and four circular plasmids. 297 Ah130 was also the only strain in our study that did not possess the cp32-8 plasmid. Cp32-8 is thought to encode multiple potential virulence factors, including a protein that enables evasion of the host immune system through the binding of factor H, a component of the host complement system [94]. Therefore, loss of the cp32-8 plasmid may contribute to the decreased infectivity of strain 297 Ah130. In addition, the two least-infectious strains, 297 Ah130 and Bb16-54, both lacked the cp32-4 plasmid, which was present in the three more infectious strains. Finally, despite previous research identifying lp25 and lp36 as “core” plasmids required for infection or correlating with increased infectivity, strains 297 Ah130 and JD1, lacking both lp25 and lp36 plasmids, were infectious in this study [86,87,88].

In this study, we have demonstrated key differences among strains of *B. burgdorferi* in female C3H/HeN mice following subcutaneous needle inoculation. Studies of variation in infection phenotype among *B. burgdorferi* strains have also been performed using a tick-infection model for enhanced ecological relevance [31,32,95,96]. It is important to note that infection phenotypes, including infectivity and tissue dissemination, differ when comparing needle inoculation and tick bite [35,36,95]. Nevertheless, needle infection remains the most common infection model of LD since it requires fewer resources to perform than tick infection. Another limitation of this study is that all the data were collected 14 days post-infection. As strains may grow at different rates, the observed discrepancies in spirochete load and pathology may be reduced or enhanced at later timepoints. Despite this consideration, examining an early timepoint provides insight into the initial dynamics of *B. burgdorferi* infection, including early spirochete dissemination, pathology, and immune activation. Considering the rising incidence of LD in the human population and the corresponding increases in research efforts, it is essential to characterize infections with model research strains and known circulating strains of *B. burgdorferi*.

## Figures and Tables

**Table 1:** Origins and ospC types of *B. burgdorferi* strains used in this study with associated references. RST: ribosomal RNA intergenic spacer type. OspC: outer surface protein C.

Strain ID	Isolate Source	Year of Original Isolation	Geographic Region	RST [39]	OspC Type	Reference(s)
297 Ah130	Laboratory isolate derived from strain 297, originally obtained from Lyme borreliosis patient cerebrospinal fluid	1982	Connecticut, U.S.	2	K	[61,62]
B31-A3	Laboratory isolate of strain B31, originally obtained from *Ixodes dammini*	1981	New York, U.S.	1	A	[63]
Bb16-54	*Ixodes scapularis*	2016	Buffalo Point, MB, CA	Unknown	I	[31,64]
Bb16-126	*Ixodes scapularis*	2016	Big Grassy, ON, CA	Unknown	N	[31,64]
JD1 (Clone SK143)	Passaged in C3H/HeN mouse, originally obtained from *Ixodes dammini*	1986	Crane’s Beach, Ipswich, MA, U.S.	3	C	[65,66]

**Table 2:** Plasmid profile of each *B. burgdorferi* strain. Presence (+) or absence (− of each plasmid was determined by PCR and gel electrophoresis for each strain tested in this study. Lp, linear plasmid. Cp, circular plasmid.

Plasmid	297 Ah130	Bb16-54	B31-A3	Bb16-126	JD1
Lp21	+	+	+	+	+
Lp28-3	+	+	+	+	+
Lp38	−	+	+	+	−
Lp28-1	+	+	+	+	+
Lp25	−	+	+	+	−
Lp36	−	−	+	+	−
Lp17	+	+	+	+	+
Lp56	−	+	+	+	−
Lp54	+	+	+	+	+
Lp28-2	+	+	+	+	+
Lp28-4	+	+	+	+	+
Lp5	−	−	−	−	−
Cp32-8	−	+	+	+	+
Cp32-1	+	+	+	+	+
Cp26	+	+	+	+	+
Cp32-4	−	−	+	+	+
Cp32-6	−	−	+	+	−
Cp32-9	−	−	+	−	−
Cp9	−	+	−	+	−
Cp32-7	+	+	+	+	+
Cp32-3	+	−	+	+	+

## Data Availability

The raw data supporting the conclusions of this article will be made available by the authors on request.

## Supplementary Materials

The following supporting information can be downloaded at https://www.mdpi.com/article/10.3390/pathogens14040352/s1: Figure S1: Plasmid profile of strain 297 Ah130; Figure S2: Plasmid profile of strain Bb16-54; Figure S3: Plasmid profile of strain B31-A3; Figure S4: Plasmid profile of strain Bb16-126; Figure S5: Plasmid profile of strain JD1. Figure S6: Serum cytokine levels following infection with B. burgdorferi strains. Serum was collected from mice 14 days after subcutaneous infection with 106 spirochetes of Bbss strain 297 Ah130, Bb16-54, B31-A3, Bb16-126, or JD1. The concentration of each cytokine was determined by a ProcartaPlex 11-plex cytokine Immunoassay kit. Error bars represent standard deviation. * *p*-value < 0.05, ** *p*-value < 0.01, *** *p*-value < 0.001, **** *p*-value < 0.0001.

## Abbreviations

The following abbreviations are used in this manuscript:

LD	Lyme disease
Bbsl	*Borrelia burgdorferi* sensu lato
Bbss	*Borrelia burgdorferi* sensu stricto
MST	Multi-locus sequence typing
osp	Outer surface protein
RST	Ribosomal RNA intragenic spacer type
BSK	Barbour–Stoenner–Kelly
PCR	Polymerase chain reaction
qPCR	Quantitative polymerase chain reaction
WBC	White blood cell
FBS	Fetal bovine serum
ELISA	Enzyme-linked immunosorbent assay
Vls	Variable major protein (VMP)-like sequence
IgG	Immunoglobulin G
IgM	Immunoglobulin M
PBS	Phosphate-buffered saline
PBS-T	Phosphate-buffered saline-Tween
Lp	Linear plasmid
Cp	Circular plasmid
