# Supplementary material for: Borrelia burgdorferi Strain-Specific Differences in Mouse Infectivity and Pathology
Source: Pathogens. 2025 Apr 5;14(4):352. doi: 10.3390/pathogens14040352 (PMC12029986; doi:10.3390/pathogens14040352)
Supplement: Supplementary file 1 [file pathogens-14-00352-s001.zip › pathogens-3545335-supplementary.pdf]

Supplementary Materials

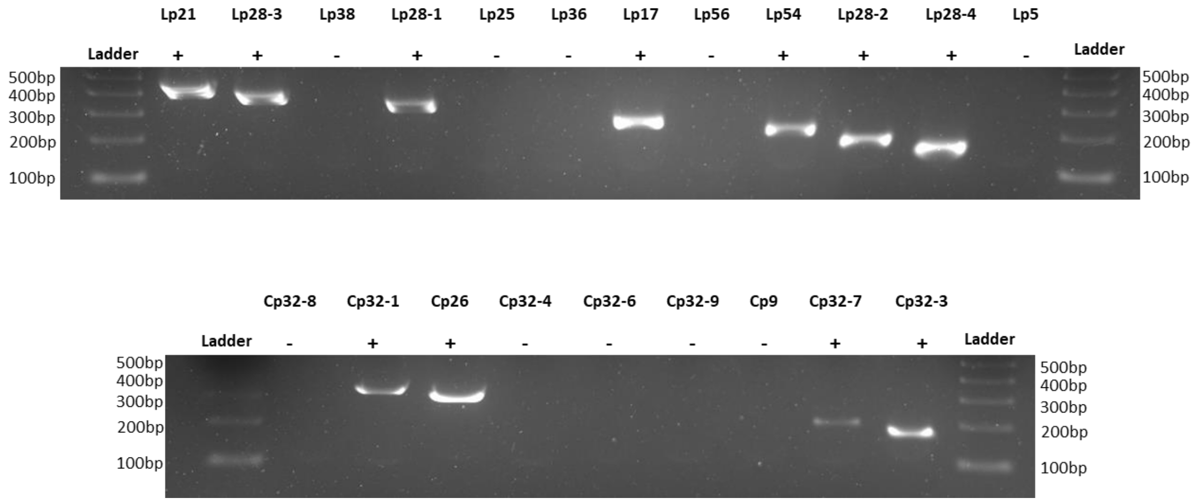

Figure S1. Plasmid profile of strain 297 Ah130. PCR amplification of genomic DNA isolated from strain 297 Ah130 was performed as described in the methods section. PCR products were separated by gel electrophoresis. The first and last lane of each gel contains a 100bp DNA ladder (Lad). Lp, linear plasmid, Cp, circular plasmid.

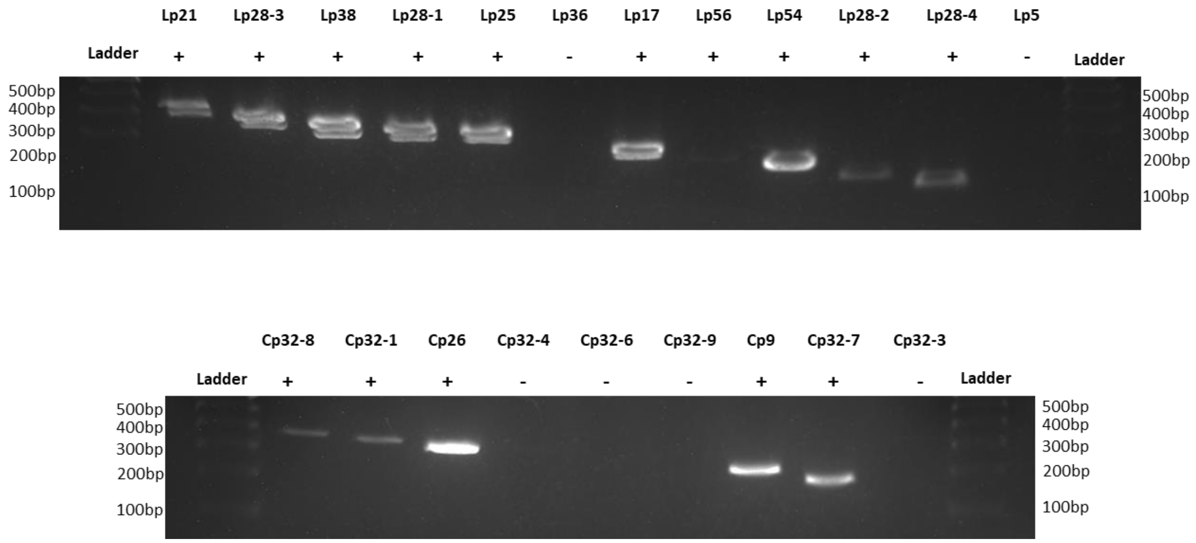

Figure S2. Plasmid profile of strain Bb16-54. PCR amplification of genomic DNA isolated from strain Bb16-54 was performed as described in the methods section. PCR products were separated by gel electrophoresis. The first and last lane of each gel contains a 100bp DNA ladder (Lad). Lp, linear plasmid, Cp, circular plasmid.

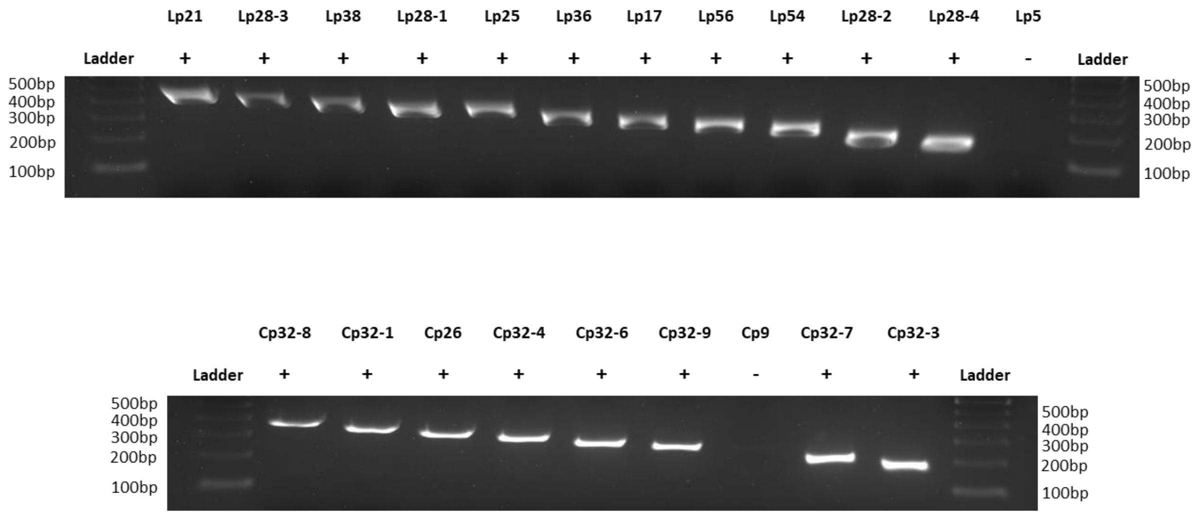

Figure S3. Plasmid profile of strain B31-A3. PCR amplification of genomic DNA isolated from strain B31-A3 was performed as described in the methods section. PCR products were separated by gel electrophoresis. The first and last lane of each gel contains a 100bp DNA ladder (Lad). Lp, linear plasmid, Cp, circular plasmid.

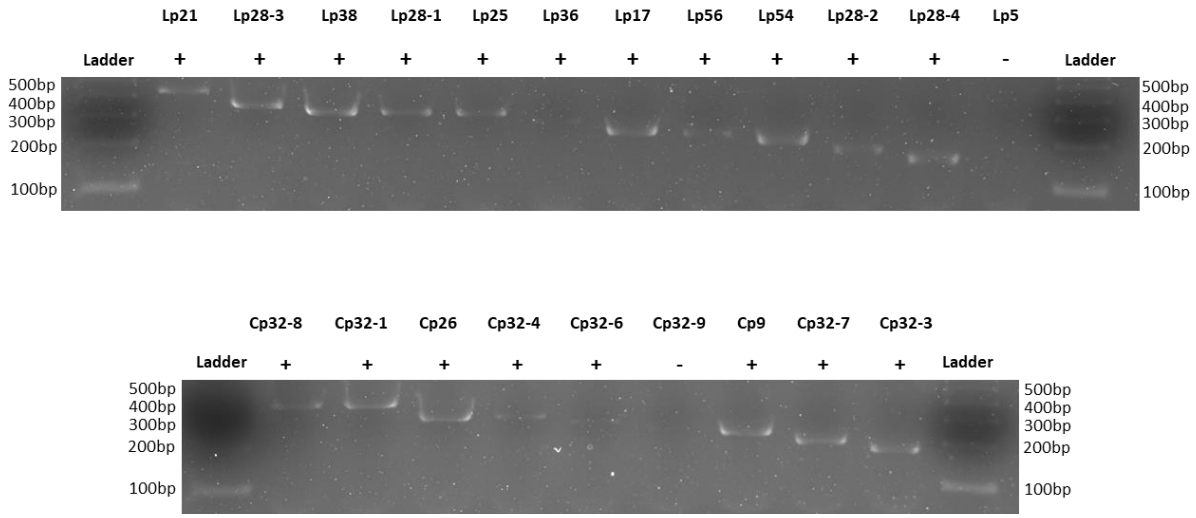

Figure S4. Plasmid profile of strain Bb16-126. PCR amplification of genomic DNA isolated from strain Bb16-126 was performed as described in the methods section. PCR products were separated by gel electrophoresis. The first and last lane of each gel contains a 100bp DNA ladder (Lad). Lp, linear plasmid, Cp, circular plasmid.

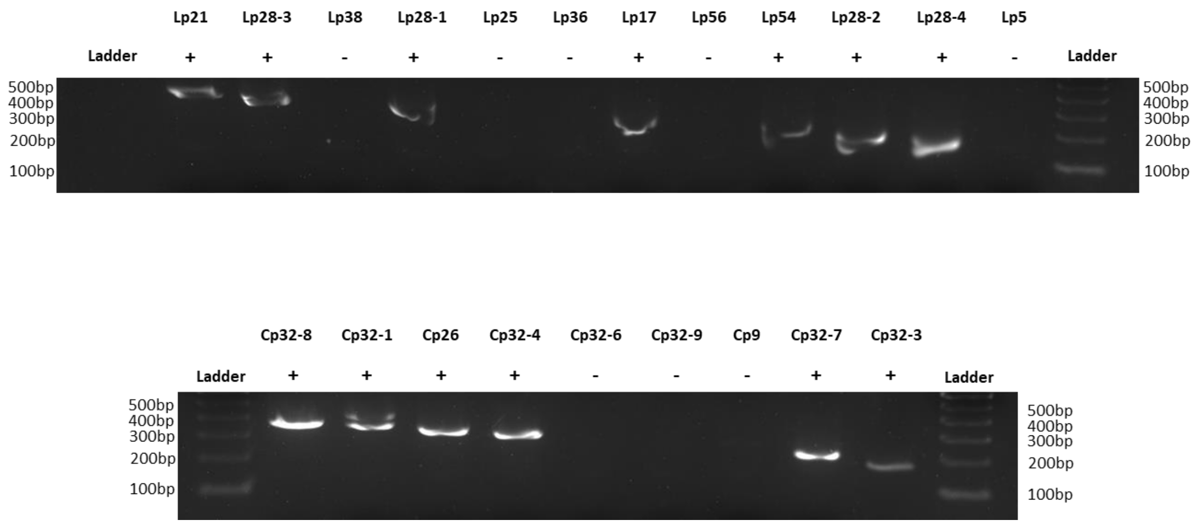

Figure S5. Plasmid profile of strain JD1. PCR amplification of genomic DNA isolated from strain JD1 was performed as described in the methods section. PCR products were separated by gel electrophoresis. The first and last lane of each gel contains a 100bp DNA ladder (Lad). Lp, linear plasmid, Cp, circular plasmid.

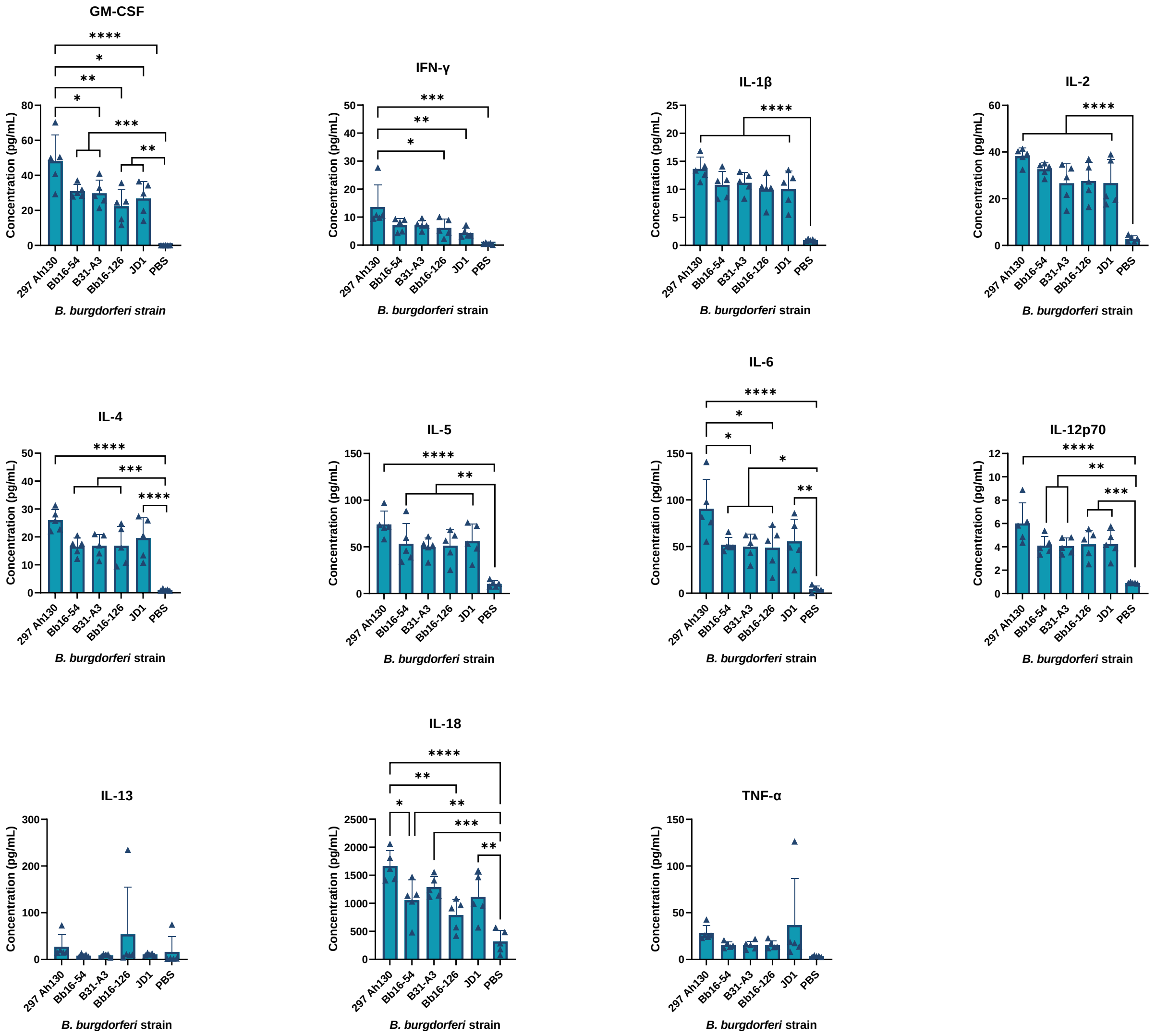

Figure S6. Serum cytokine levels following infection with *B. burgdorferi* strains. Serum was collected from mice 14 days after subcutaneous infection with 10^6 spirochetes of Bbss strain 297 Ah130, Bb16-54, B31-A3, Bb16-126, or JD1. The concentration of each cytokine was determined by a ProcartaPlex 11-plex cytokine Immunoassay kit. Error bars represent standard deviation.* p-value < 0.05, ** p-value < 0.01, *** p-value < 0.001, **** p-value < 0.0001.
